# Supplementary material for: Effects of Socio-Environmental Factors on Malaria Infection in Pakistan: A Bayesian Spatial Analysis
Source: Int J Environ Res Public Health. 2019 Apr 16;16(8):1365. doi: 10.3390/ijerph16081365 (PMC6517989; doi:10.3390/ijerph16081365)
Supplement: Supplementary file 1 [file ijerph-16-01365-s001.pdf]

Supplemental materials

Table S1. Descriptive statistics of malaria annual incidence (per 100,000) and socio-environmental variables at the district level in Pakistan, 2013, 2014 and 2015.

Year	Variables	Mean	Std. Deviation	Minimum	Quantiles			Maximum
					25	50	75	
2013	Incidence	609.86	1885.74	0.00	6.57	112.52	663.12	19,018.40
	HDI	0.46	0.13	0.13	0.38	0.48	0.56	0.71
	ES	60.89	14.09	29.44	49.99	62.17	72.82	84.85
	PD	313.54	438.27	4.00	57.00	224.50	444.50	3566.00
	RF	474.69	332.20	122.83	191.98	389.88	654.72	1409.51
	T (min.)	17.02	2.99	8.74	15.35	17.68	19.25	21.47
	T (max.)	30.37	2.75	23.93	28.32	31.26	32.64	33.53
	T (mean)	23.69	2.84	16.33	21.84	24.53	25.87	27.46
	RH	60.07	4.68	47.79	58.27	61.06	63.80	66.21
	WS	3.14	1.69	1.60	1.96	2.31	3.69	8.32
2014	Incidence	565.73	1379.49	0.00	5.16	118.13	589.69	12,442.91
	HDI	0.46	0.13	0.13	0.38	0.48	0.56	0.71
	ES	60.89	14.09	29.44	49.99	62.17	72.82	84.85
	PD	313.54	438.27	4.00	57.00	224.50	444.50	3566.00
	RF	462.85	323.29	56.11	171.12	387.59	697.07	1213.48
	T (min.)	15.46	3.52	7.81	12.89	15.45	17.93	23.13
	T (max.)	28.99	2.68	23.26	27.22	28.98	30.65	34.12
	T (mean)	22.23	3.06	15.53	20.05	22.14	24.28	28.63
	RH	52.46	6.32	34.50	48.36	54.20	57.57	60.95
	WS	3.65	1.66	1.98	2.31	2.86	4.88	8.20
2015	Incidence	390.51	753.32	0	3.73	85.13	437.34	4335.28
	HDI	0.46	0.13	0.13	0.38	0.48	0.56	0.71
	ES	60.89	14.09	29.44	49.99	62.17	72.82	84.85
	PD	313.54	438.27	4	57	224.5	444.5	3566
	RF	607.67	364.39	124.14	276.75	501.64	854.3	1619.6
	T (min.)	16.04	3.52	7.98	13.5	16.03	18.39	23.86
	T (max.)	29.13	2.9	22.36	27.31	29.17	31.1	34.43
	T (mean)	22.58	3.15	15.17	20.22	22.54	24.65	29.15
	RH	54.97	7.21	32.87	51.3	56.58	60.92	63.73
	WS	4.01	1.47	2.59	2.81	3.33	5.06	8.23

Note: Each variable in the table represents annual average for 136/146 districts in Pakistan.
HDI (human development index), ES (education score), PD (population density, per km^2), RF (rainfall, mm), T (min.) (minimum temperature, °C), T (max.) (maximum temperature, °C), T (mean) (mean temperature, °C), RH (relative humidity, %), WS (wind speed km/h).

Table S2. Spearman's correlation between malaria incidence and socio-environmental variables at the district level in Pakistan, 2013, 2014 and 2015.

Year	Variables	Incidence	HDI	ES	PD	RF	T (min.)	T (max.)	T (mean)	RH	WS
2013	HDI	−0.722 **	1								
	ES	−0.671 **	0.677 **	1							
	PD	−0.627 **	0.518 **	0.686 **	1						
	RF	−0.380 **	0.283 **	0.512 **	0.376 **	1					
	T (min.)	0.237 **	−0.141	−0.340 **	−0.226 **	−0.950 **	1				
	T (max.)	0.411 **	−0.298 **	−0.505 **	−0.374 **	−0.975 **	0.949 **	1			
	T (mean)	0.331 **	−0.226 **	−0.438 **	−0.308 **	−0.974 **	0.987 **	0.984 **	1		
	RH	−0.298 **	0.164	0.527 **	0.421 **	0.846 **	−0.749 **	−0.791 **	−0.787 **	1	
	WS	−0.050	0.164	−0.085	−0.080	−0.732 **	0.793 **	0.692 **	0.757 **	−0.742 **	1
2014	HDI	−0.709 **	1								
	ES	−0.628 **	0.677 **	1							
	PD	−0.604 **	0.518 **	0.686 **	1						
	RF	−0.265 **	0.273 **	0.525 **	0.377 **	1					
	T (min.)	−0.120	0.048	0.045	0.138	−0.669 **	1				
	T (max.)	0.039	−0.095	−0.182 *	−0.080	−0.827 **	0.950 **	1			
	T (mean)	−0.048	−0.021	−0.064	0.040	−0.750 **	0.988 **	0.985 **	1		
	RH	−0.464 **	0.401 **	0.731 **	0.604 **	0.768 **	−0.075	−0.323 **	−0.193 **	1	
	WS	0.210 *	−0.135	−0.462 **	−0.425 **	−0.908 **	0.497 **	0.663 **	0.578 **	−0.779 **	1
2015	HDI	−0.713 **	1								
	ES	−0.626 **	0.677 **	1							
	PD	−0.589 **	0.518 **	0.686 **	1						
	RF	−0.284 **	0.237 **	0.497 **	0.379 **	1					
	T (min.)	−0.024	0.021	−0.004	0.077	−0.671 **	1				
	T (max.)	0.213 *	−.0193 *	−0.311 **	−0.206 *	−0.835 **	0.919 **	1			
	T (mean)	0.085	−0.079	−0.145	−0.049	−0.754 **	0.983 **	0.973 **	1		
	RH	−0.306 **	0.236 **	0.617 **	0.495 **	0.792 **	−0.159	−0.425 **	−0.277 **	1	
	WS	0.251 **	−0.163	−0.497 **	−0.462 **	−0.948 **	0.555 **	0.758 **	0.651 **	−0.834 **	1

Note: ** Correlation is significant at the 0.01 level (2-tailed), * Correlation is significant at the 0.05 level (2-tailed). Each variable in the table represents annual average for 136/146 districts in Pakistan. HDI (human development index), ES (education score), PD (population density, per km^2), RF (rainfall, mm), T (min.) (minimum temperature, °C), T (max.) (maximum temperature, °C), T (mean) (mean temperature, °C), RH (relative humidity, %), WS (wind speed km/h).

Table S3. Association between socio-environmental factors and malaria incidence in Pakistan, 2013, 2014 and 2015 (Bayesian CAR model).

Year	DIC	Variable	Mean	SD	MC error	2.50%	Median	97.50%
2013	1344.42	HDI	−5.477	1.62	0.08671	−8.673	−5.134	−2.861
		ES	−0.00525	0.01262	6.76×10^{-4}	−0.0329	−0.003782	0.01473
		PD	-7.00×10^{-4}	3.75×10^{-4}	1.88×10^{-5}	−0.001377	-7.26×10^{-4}	8.56×10^{-5}
		RF	0.001797	0.001694	9.08×10^{-5}	-6.89×10^{-4}	0.001598	0.006139
		T (min.)	0.397	0.1694	0.009111	0.06315	0.4121	0.6559
		RH	−0.05364	0.03332	0.001791	−0.09467	−0.06589	0.005503
2014	1340.03	HDI	−4.448	1.526	0.08153	−7.555	−4.473	−1.658
		ES	−0.01417	0.0178	9.55×10^{-4}	−0.05111	−0.01042	0.01342
		PD	-5.79×10^{-4}	3.98×10^{-4}	2.04×10^{-5}	−0.001287	-5.97×10^{-4}	1.95×10^{-4}
		RF	0.001886	9.27×10^{-4}	4.94×10^{-5}	-2.23×10^{-4}	0.001911	0.003854
		T (min.)	0.1191	0.06091	0.003268	0.03621	0.1007	0.2593
		RH	0.007465	0.00922	4.92×10^{-4}	−0.009674	0.007725	0.02358
2015	1296.66	HDI	−4.542	1.634	0.08719	−7.52	−4.592	−1.523
		ES	−0.02885	0.02462	0.001323	−0.06598	−0.02434	0.01737
		PD	-7.08×10^{-4}	4.22×10^{-4}	2.12×10^{-5}	−0.001503	-7.31×10^{-4}	1.10×10^{-4}
		RF	4.40×10^{-4}	0.001068	5.71×10^{-5}	−0.001206	2.76×10^{-4}	0.002609
		T (min.)	0.1373	0.04801	0.00257	0.01537	0.1446	0.2047
		RH	−0.04485	0.01855	9.95×10^{-4}	−0.09003	−0.04403	−0.009491

Note: Each variable in the table represents annual average for 136/146 districts in Pakistan. DIC (deviance information criteria), SD (standardized division), HDI (human development index), ES (education score), PD (population density, per km^2), RF (rainfall, mm), T (min.) (minimum temperature, °C), RH (relative humidity, %).

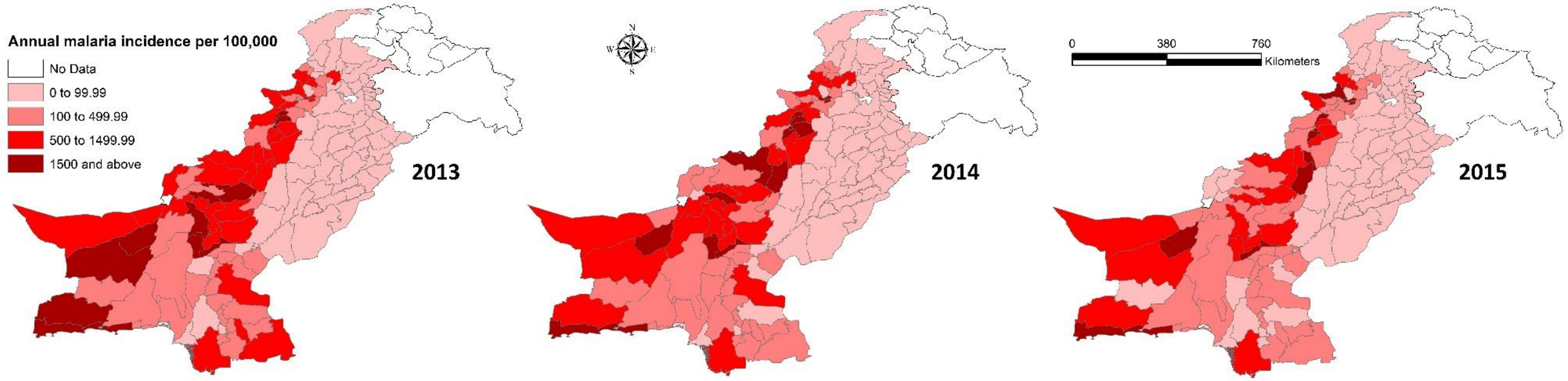

Figure S1. Annual malaria incidence 2013, 2014 and 2015.

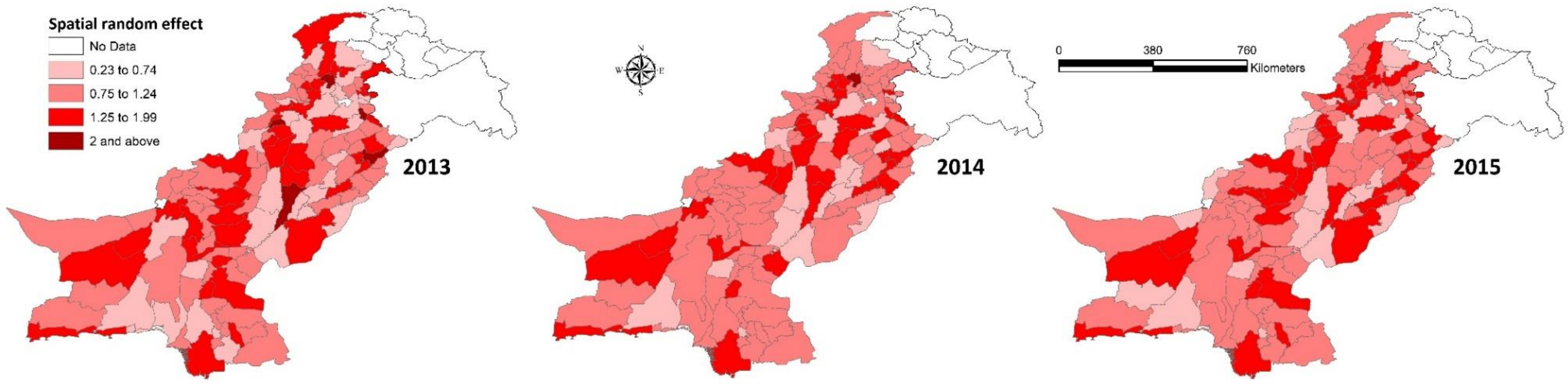

Figure S2. Structured spatial random effects 2013, 2014 and 2015.
